# Perovskite Puzzle for Revolutionary Functional Materials

**DOI:** 10.3389/fchem.2020.550625

**Published:** 2020-11-02

**Authors:** Nikolai Belich, Natalia Udalova, Anna Semenova, Andrey Petrov, Sergey Fateev, Alexey Tarasov, Eugene Goodilin

**Affiliations:** ^1^Department of Materials Science, Lomonosov Moscow State University, Moscow, Russia; ^2^Department of Chemistry, Lomonosov Moscow State University, Moscow, Russia

**Keywords:** perovskites, superconductivity, magnetoresistance, photovoltaics, copper, manganese, perovskite solar cells, lead halides

## Abstract

Widely spread crystal lattices of perovskites represent a natural flexible platform for chemical design of various advanced functional materials with unique features. An interplay between chemical bonding, defects and crystallochemical peculiarities makes the perovskite structure a “LEGO designer” utilizing natural features of chemical elements of the renowned Mendeleev's Periodic Table (PTE) celebrating its 150-year anniversary. In this mini-review, crystal chemistry and bonding features, physical and functional properties, preparation methods and tuning functional properties with periodicity “tools” of the PTE will be exemplified for legendary families of high-temperature superconductive cuprates, colossal magnetoresistive manganites and hybrid lead halides for a new generation of solar cells.

## Introduction

**Figure 1:**
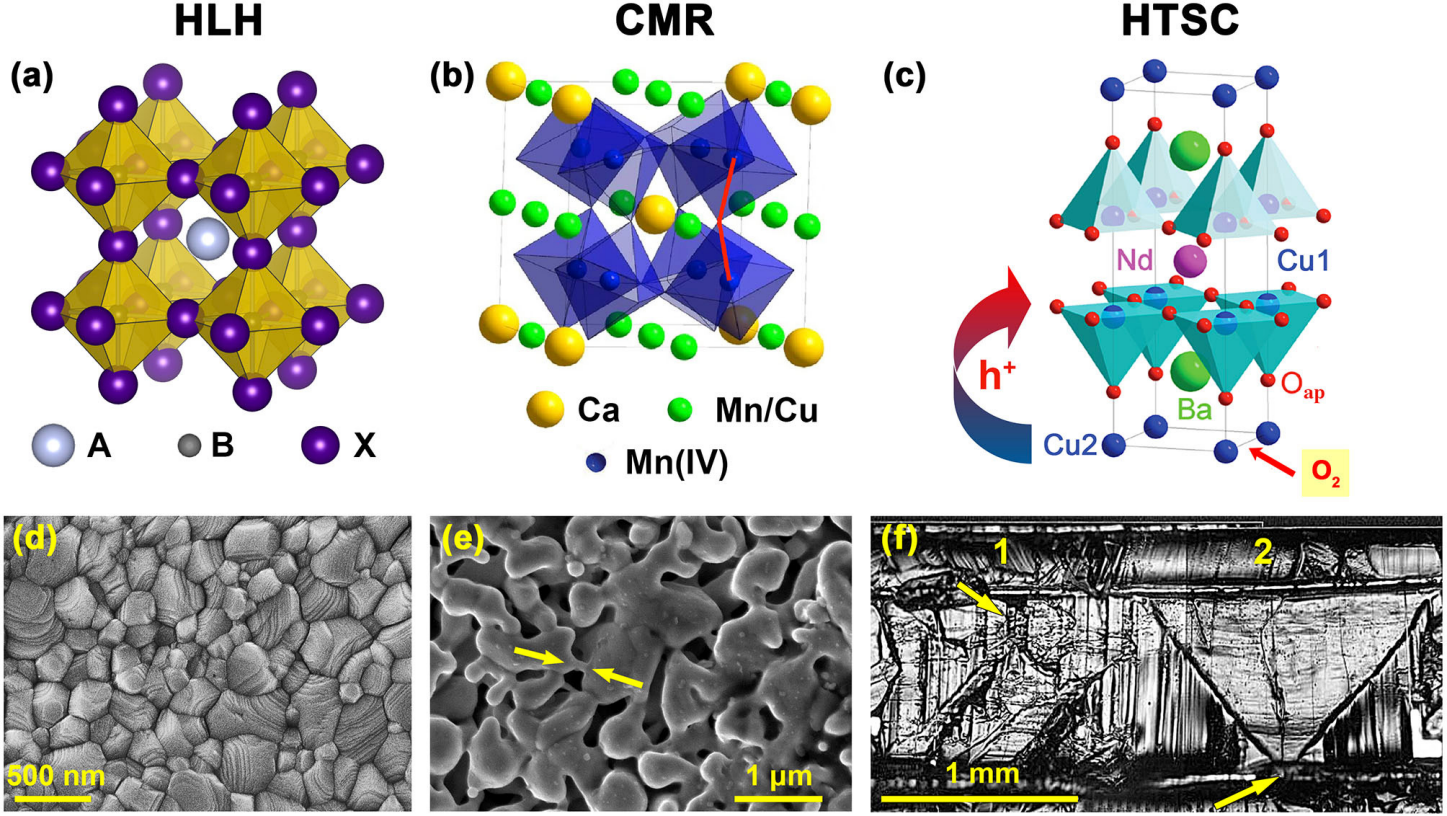
Crystal structures **(a,b,c)** and, respectively, typical morphologies from a top view of films or coatings of solution–derived HLH perovskites **(d)**, ASP—deposited CMR manganites **(e)** and melt—processed HTSC cuprates **(f)**. The A, B, X for the structure of HLH perovskite **(a)** represent a large inorganic or organic cation (A), lead (B) and halogen (X), respectively. The structure of CMR manganites **(b)** is shown for a special family of the CaCu_3_Mn_4_O_12_ solid solution. The HTSC is represented by the most famous family of REE-barium cuprates (NdBa_2_Cu_3_O_6+x_), the arrow symbolizes the hole (h^+^) transfer from Cu(2) ions in the charge reservoir to Cu(1) in superconducting plains. The white arrows show intergrain necks **(e)** or nucleation sites of crystallization for two adjacent millimeter–sized HTSC grains on a graphotexturing substrate **(f)**. Note, the morphology shown for CMR manganites is not acceptable for HLH or HTSC perovskites while the HLH microstructure would not be suitable for HTSCs.

The perovskite lattice (Figure 1) represents one of the most common motifs of solid phases and, also, is quite typical for the famous families of advanced functional materials including photocatalysis, electrocatalysts and fuel cell components, insertion cathodes of chemical power sources, high—temperature superconductors, multyferroics, magnetic and magnetoresistive materials, materials for solar cell energy and photoluminescence (Ahn et al., 2004; Haugan et al., 2004; Gao et al., 2010; Du et al., 2011, 2013; Jiang et al., 2012; Osterloh, 2013; Frost et al., 2014; Song et al., 2015; De Roo et al., 2016; Weidman et al., 2016; Hwang et al., 2017, 2019; Wang et al., 2019; Hao et al., 2020). At the same time only three families of perovskite-based functional materials have attracted major interest in view of prospects in developing mass-production technologies and practical applications.

**C**olossal **M**agneto**R**esistive (CMR) manganites (Figure 1b) were discovered in the 1950's (Van Santen and Jonker, 1950; Volger, 1954) and rediscovered in the 1990's (von Helmolt et al., 1993). In 2007, a Nobel Prize in Physics was awarded for the same effect observed in layered heterostructures demonstrating the Giant MagnetoResistance based on the fundamentally important effect of control of electron scattering by spin polarization (Fert et al., 1995). **H**igh–**T**emperature **S**uper **C**onducting (HTSC) cuprates (Figure 1c) have been found in 1986 (Bednorz and Müller, 1986) and have been awarded next year with the Nobel Prize in Physics which is one of a total of six Nobel Prizes for superconductivity. The most recent high-impact perovskites (Figure 1a) have been suggested for **P**erovskite **P**hotovoltaics presented solely by **H**ybrid **L**ead **H**alides applied for solar cells in 2009 by Tsutomi Miyasaka (Kojima et al., 2009), developed further in 2012 by Michael Grätzel with essential works known from Henry Snaith, Nam-Gyu Park, and others (Grätzel, 2014; Park et al., 2016; Eperon et al., 2017; Li et al., 2017, 2018; Gao et al., 2018; Leijtens et al., 2018; Snaith and Hacke, 2018; Fu et al., 2019; Nayak et al., 2019). They represent a new generation of solar cell materials overcoming easily the records of DSSC devices with chemically stable and quite effective tetrapyrolic sensitizers and approaching closely the best values of effectiveness of silicon (Bottari et al., 2010; Li and Diau, 2013; Mathew et al., 2014; Martynov et al., 2019; Abdulaeva et al., 2020).

These families of perovskite-like materials represent three conceptually different areas of materials research for advanced electronics and alternative energy. The unique behavior of these materials originates of chemical element features composing the phases, different metal—non-metal bonds resulting in different target physical properties and demanding, in turn, quite special optimal morphologies of the materials under the question (Figure 1). The latter, obviously, can be achieved by material-specific production techniques being the limiting factors of the materials implementation. It is risky to compare all these almost dissimilar materials (Table 1) but there is the only valuable aspect of such a story that is closely related to the key chemical approaches utilized for enhancing their optimal morphologies. This material—related analysis seems to be useful for a rational design and future progress in preparation techniques toward the development of the perovskite—based devices with record characteristics. This purpose is highlighted in the current mini-review to shape the research directions of prospective chemical preparation routes based on the PTE peculiarities of the respective elements.

**Table 1:** Basic features of HTMC cuprates, CMR manganites and HLH solar perovskites in comparison.

**Features**	**HTSC cuprate perovskites**	**CMR manganite perovskites**	**Solar/hybrid lead halide perovskites**
Key chemical elements configuration, ion size and framework bonding	Cu—O (d/p) Cu: [Ar]3d^10^4s^1^ O: [He]2s^2^2p^4^ 68−71 pm for Cu(III)—Cu(I) 126 pm for O^2−^	Mn—O (d/p) Mn: [Ar]3d^5^4s^2^ O: [He]2s^2^2p^4^ 67−72 pm for Mn(IV)—Mn(III) 126 pm for O^2−^	Pb—I (s/p, p/p) Pb: [Xe]4f^14^5d^10^6s^2^6p^2^ I: [Kr]4d^10^5s^2^ 5p^5^ 133 pm for Pb(II) 206 pm for I^−^
Functional properties	Superconductivity Diamagnetic	Magnetoresistance Magnetic semiconductor / metallic	Photoeffect Semiconductor
Carriers	Hole pairs (bosons, BCS pairs)	Electrons (spin–polarized)	Hole–electron pairs (excitons)
Metal oxidation state(s)	Mixed +2/+3	Mixed +3/+4	Fixed +2
Conduction path	Flat CuO_2_ sheets (doped from charge reservoir)	Lined Mn–O–Mn chains (double exchange etc.)	Pb–I–Pb chains (“redox”)
Point defects	Disordered and ordered oxygen vacancies, cation antisites, homo- and heterovalent substitution in both cation and anion sublattices	Disordered oxygen vacancies, cation antisites, homo- and heterovalent substitution in both cation and anion sublattices	Mostly homovalent substitution in either cation or anion sublattices, iodine vacancies
Deviation from stoichiometry ratio	Large for oxygen, much smaller for the larger central cations and copper (wide range for proper substitutions)	Small for both oxygen and cations (wide range for proper substitutions)	Iodine stoichiometry (still unclear) (wide range for proper substitutions)
Carrier generation	Oxidation	Heterovalent substitutions	Light absorption
Local distortions	Jahn–Teller effect, ion mismatch	Jahn–Teller effect, ion mismatch	Ion mismatch
Microstructure required	Biaxial texturing, large grains, clean boundaries, no weak links	Intergrain tunneling (other requirements are not essential)	No pinholes, no charge traps at grain boundaries, large grains are better, no texture is required
Whiskers	Exist, no need	Exist, no need	Exist, possibly useful
Applications	Large grain ceramics, epitaxial thin films, heterostructures	Thin films, polycrystalline coatings	Polycrystalline thin films, heterostructures, quantum dots, single crystals
Best processing	Melt techniques (LAP, MTG, LPP, PDMG, IMC, GPM, CGMG, SLMG, PMP, TPP, GEORGE, QMG, OCMG, MPMG, QDR) and thin films (ASP/CVD/MOCVD/PVD/RaBiTS)	Ceramic sintering, thin films (CVD/MOCVD/PVD/ASP)	Thin films (solution/precipitation, CVD/PVD/ASP, RP-MAGIC)
Spinodal decomposition	Known, useful for pinning	Known, useless	Known, under study

*LAP, liquid assisted processing (crystallization or recrystallization with traces of melt); MTG, melt textured growth (melting and cooling process under constant pO_2_); LPP, liquid phase processing (melting and stepwise cooling); PDMG, platinum doped melt growth; IMC, isothermal melt crystallization (melting and crystallization by pO_2_ variation under constant temperature); GPM, gas pressure method (crystallization under elevated partial pressure of oxygen); CGMG, constant gradient melt growth (crystallization along the concentrational/spatial gradient of REE); SLMG, solid liquid melt growth (melting and cooling process of fine mixture of powders under constant pO_2_); PMP, powder melt process; TPP, two powder process; GEORGE, GEometrically-ORganized-Growth-Evaluation (crystallization along the geometrically created concentrational / spatial gradient of REE); QMG, quench melt growth; OCMG, oxygen controlled melt growth; MPMG, melt powder melt growth; QDR, quenched directional recrystallization; RP-MAGIC, reactive polyiodide melt assisted growth through in situ conversion; (MO) CVD, (metal-organic) chemical vapor deposition; PVD, physical vapor deposition; ASP, aerosol spray pyrolysis*.

## Structural Features of Perovskites vs. Composing Element Peculiarities

None of the discussing materials demonstrate ideal ABX_3_ perovskite structures where A—a larger central cation, B—a smaller cation octahedrally surrounded by X anions (Figure 1). Moreover, different HTSC, CMR, HLH families and homologs, intergrown or none-perovskite structures are well-known (Zhang et al., 1996; Tretyakov and Goodilin, 2000; Tretyakov et al., 2004; Attfield, 2011; Lee et al., 2014; Ovcharov et al., 2019). However, their “classical” representatives are stack to the simplest perovskite lattice (Table 1, Figure 1). HTSC cuprates and CMR manganites, according to the Pearson's formalism^1^, are the compounds of hard acids (cations) from the d-block (Cu and Mn) and a hard base O^2−^ from the p-block. On the contrary, the lead halide perovskites are composed from a soft acid Pb^2+^ and a soft base I^−^ from the p-block of PTE. These combinations result in phases which are more stable but different. The hard acid–hard base compounds with stronger interactions of smaller non-polarizable ions in the lattice exist in air up to the temperatures of ca. 1000 (HTSC)−1,300°C (CMR) while the HLH soft acid–soft base perovskites with large polarizable ions do not survive, expectedly, above 120–150°C but easily form solutions and adducts with various donor solvents (Fateev et al., 2018) thus presenting the most attractive solvent-based deposition technologies of solar cell production (Park, 2016). HLH is a unique compound family made of the heaviest and the largest non-radioactive elements of PTE.

HTSC cuprates (Figure 1c) and CMR manganites (Figure 1b) are mixed**—**valent phases with a large non-stoichiometry of X**—**anion for HTSC or A**—**cation in the case of CMR phases (Tretyakov and Goodilin, 2000; Pomerantseva et al., 2004; Tretyakov et al., 2004). The HLH perovskites demonstrate a moderate or small X-site non-stoichiometry and, formally, fixed oxidation states of lead and halogens. The mixed-valent states of HTSC cuprates and CMR manganites are achieved by means of two chemically different approaches. Oxygen non-stoichiometry is the major factor used for oxidation of the HTSC phases with molecular oxygen (Figure 1) and that leads to an increase of the copper oxidation state, for example, from +1/+2 for REEBa_2_Cu_3_O_6_ (REE**—**Rare Earth Elements) to +2 and exotic Cu(III) for REEBa_2_Cu_3_O_7_ (Shiohara and Goodilin, 2000; Tretyakov and Goodilin, 2000). CMR manganites use heterovalent doping of the A**—**cation rather than varying oxygen content to provide the needed balance of Mn(III) / Mn(IV) in the structure. Normally, the B**—**site substitution, especially heterovalent, provides no drastic improvement of functional properties and, often, deteriorates them. For example, <1–5 at % of Zn, Mg, and other elements substituting copper in REEBa_2_Cu_3_O_7_ lead to a half reduction of the superconductivity transition temperature, similar substitutions of manganese in CMR manganites are also risky, have no linear dependence on their concentration and normally are not effective for properties improvements. For HLH (Figure 1a), lead substitution with tin, bismuth etc. usually results in decreasing functional parameters while the X-site substitution with mixed halide ions is often useful for fine tuning of the physical and thermodynamic properties. The X-site substitution, even heterovalent (like fluorine), is applied rarely to tune the properties of HTSCs and CMR materials but it could not be considered as primary method of their target chemical modifications.

The A-site cation plays an important but a secondary role. The A-cation usually affects no physical properties but it is primarily needed to stabilize the structure electrostatically and geometrically since the ionic radii of this cation is counted in the famous Goldshmidt tolerance factor predicting the overall structure stability. Noticeably, a 12–13% decrease of the REE size due to the lanthanide contraction effect results in the REEBa_2_Cu_3_O_7_ melting temperature falling by about 120°C (Shiohara and Goodilin, 2000), from 1,085°C (Nd) down to 965°C (Yb); this effect is much weaker for mixed REE–AEE CMR manganites (AEE**—**Alkali Earth Elements). In the case of HLH, the largest purely inorganic cation in the PTE, Cs^+^, seems to be still too small to solely stabilize the HLH perovskite structure near room temperature thus demanding a larger cation, such as methylammonium and formamidinium (Travis et al., 2016). The latter makes the HLH perovskites belong to the hybrid, organic–inorganic, phases and therefore this feature entirely changes their chemical properties and preparation techniques. A further increase of the A-cation size or changing its geometry produces low-dimensional HLH phases with complex structures (Smith et al., 2018); thus a set of available cations to form the HLH perovskites is quite limited. The consequences of the asymmetry of such a “hybrid” cation include local structural distortions and, for some solid solutions, a possibility of spinodal decomposition which is useless for the HLH perovskites. Oppositely, the spinodal decomposition is a remarkable phenomenon for the A-site substituted solid solutions based on the HTSC cuprates (Petrykin et al., 2000; Shiohara and Goodilin, 2000) since the demixing generates compositional nanofluctuations acting as effective pinning centers and thus resulting in record critical currents under magnetic fields. A more complex structure of HTSCs and CMR manganites could also yield antisites in the structure of perovskites resulting in the preparation “prehistory” effects (Petrykin et al., 2000). In the case of HLH perovskites, a mixture of various A-cations are routinely applied for the entropy-driven stabilization (Yi et al., 2016).

The framework of corner-sharing octahedra BX_6_ of the perovskites generates the main application-related properties of these materials (Tretyakov et al., 2004). In this contest, The Jahn–Teller effect, being quite typical for Mn^3+^ and Cu^2+^, causes frustrated structures, spin waves for manganites (Pomerantseva et al., 2004) or result in drastic structural distortions for HTSCs. In the case of REEBa_2_Cu_3_O_7_, the structure (Figure 1c) is composed of three perovskite-like oxygen deficient intergrown blocks (Shiohara and Goodilin, 2000; Tretyakov and Goodilin, 2000). The two of them containing barium and empty oxygen vacancies near copper (Cu(1)O_2_**—**BaO_(ap)_**—**Cu(2)O_x_) operate as charge reservoirs accumulating holes upon copper oxygenation and oxygen content growth, V_O_^**^ + 1/2 O_2_ = O_O_^X^ + 2 h^*^. It is a direct representation of the Jahn–Teller effect that there is no octahedra with copper and oxygen in this structure but, instead, Cu(1) is included into flat superconducting (SC) planes CuO_2_ and have a five-fold pyramidal coordination counting also the “apical” oxygen O_(ap)_ in the BaO “layer.” The second type of copper, Cu(2), possesses a two-fold linear coordination for the tetragonal oxygen-disordered REEBa_2_Cu_3_O_6_ compound or rhombs for the superconducting REEBa_2_Cu_3_O_7_ orthorhombic phase with ordered residual oxygen vacancies. The central CuO_2_-REE–CuO_2_ block contains two flat superconducting planes and REE^3+^ cations. Upon oxygenation, holes are concentrated in the charge reservoir blocks and are transferred then to the superconducting plains CuO_2_ by shifting the apical oxygen O_(ap)_ from Cu(2) toward Cu(1). A critical concentration of holes in the SC plains gives bosons, the BCS pairs, if cooled below a SC critical temperature T_c_. Actually, such a crystal architecture makes HTSCs cuprates highly anisotropic layered compounds originated of perovskites.

The flatter the SC plains, the higher T_c_ of HTSCs, otherwise overlapping the d-orbitals of Cu(1) and p-orbitals of oxygen is deteriorated. In the case of CMR manganites and HLH perovskites, no deep modifications of the perovskite motif are observed (Tretyakov et al., 2004). Moreover, the Mn–O–Mn chains in the structure of CMR phases (Figure 1b) have to be linear with the angle between Mn^3+^, O^2−^ and Mn^4+^ close to 180° for effective overlapping of the respective d_Mn_ and p_O_ orbitals corresponding each other by symmetry (Babushkina et al., 1998; Pomerantseva et al., 2004). Due to the antiferromagnetic, double exchange in these linear fragments, electrons may transfer from Mn^3+^ to Mn^4+^ via the linking oxygen. To reduce the electrical resistance of the phase, the electron carriers should be correlated or spin–polarized by an external magnetic field in different parts of CMR manganite and grains which results in the negative magnetoresistance effect. The overlapping of the s, p-orbitals of iodine and lead in HLH semiconducting phases also provides effective pathways for charge transfer however both hole and electron carriers are generated by the photovoltaic effect utilizing electron density redistribution between the s- and p–orbitals of Pb^2+^ and p–orbitals of I^−^ within the Pb-I-Pb framework (Table 1). The HLH phases seem to be mostly “tolerant” to various defects (Meggiolaro et al., 2018) as not typical for classical semiconductors. Among others, the HLH perovskites seem to be the chemically and physically simplest phases (Figure 1a) with no peculiarities caused by defect ordering, heterovalent substitution or spin-correlated phenomena. The noted peculiarities of the discussed frameworks pre-determine, to a large extent, both the morphology and production schemes of the final materials and devices.

## Morphologies, Microstructures and Relevant Processing Techniques

Nowadays, the perovskite-like phases under discussion are mostly used as 2D polycrystalline materials (thin films and heterostuctures) with already rare inventions of their 3D ceramic or single crystalline forms. As a polycrystalline matter for advanced practical applications, each of the materials requires its own optimal combination of general morphological parameters like crystalline grain size, orientation, thickness, uniformity and the organization of intergrain boundaries since the required different morphologies of the materials ensure the achievement of record functional properties. All the HTSCs, CMR and HLH materials are prominent in low-current applications, sensing devices and smart circuits for information technologies, communication, and microelectronics. HTSCs and CMR materials are involved in applications as magnetic field sensors, in both the cases, the artificial or natural organization of grain boundaries play an extraordinary role. The most known and the most magnetic field sensitive devices are SQUID magnetometers and tomographs utilizing the quantum Josephson's effect for a special gap / boundary architecture within a superconductor (Colclough et al., 1987). CMR materials for spintronics utilize artificial junctions to operate with spin-polarized carriers created by a magnetic field in spin valves and other elements of spintronics (Yang et al., 2019); in the simple case of magnetic sensors, grain boundaries within CMR manganites play a major role for target variation of electrical conductivity. It should be noted that, oppositely, multiple grain boundaries play a negative role for HTSCs even in those low-current applications. HLH semiconducting materials demonstrate a photovoltaic effect leading to generation of carriers for conversion of solar energy into electricity (Grätzel, 2014; Chen et al., 2017; Eperon et al., 2017). In such a case, grain boundaries are not a positive factor since they could quench and trap the carriers reducing the operational effectiveness although they are not really used for the control of a transport current. Vice versa, HLH materials can effectively generate light by converting electrical energy in light-emitting devices and quantum dots under low voltages and low currents (De Roo et al., 2016; Fu et al., 2019). HTSCs are the only type of materials in this group requiring high current applications demanded for industrial transport of electricity, industrial current limiters or generation of record magnetic fields in energy generation or transport systems (Bednorz, 2019; Dong et al., 2019).

Thus, HTSCs, CMR manganites, HLH perovskites demand three different approaches to achieve a proper morphology (Figure 1). For important devices utilizing these three perovskites, thin films, heterostructures or sandwiched structures have to be deposited (Zhang et al., 1996; Shiohara and Goodilin, 2000; Tretyakov and Goodilin, 2000; Tretyakov et al., 2004; Snaith and Hacke, 2018). CMR manganites (Pomerantseva et al., 2004) operate with a relative change of resistance under applied magnetic fields and this demands lateral transport of weak electrical currents while intergrain boundaries become highly important due to the effect of tunneling magnetoresistance (TMS). This means that CMR manganites would require a uniform thickness of the films but not necessarily their single crystallinity, polycrystalline CMR films seem to have some advantages thus this type of perovskites post the weakest requirements to their microstructure (Figure 1e). HTSC cuprate films below 1 μm in thickness, preserving the epitaxial control over the biaxial texturing, are necessary for spreading a lateral critical current for long distances (RABiTS tapes, rolling-assisted-biaxially-textured-substrate, and other “second generation” HTSCs) but its values exceed a fantastic level of 10^7^ A/cm^2^ which is achievable by no other known materials. Under such a large current, non-uniformity of any kind or “weak links” between the grains result immediately in “hot spots” with huge local overheating leading finally to thermal destruction of the films. Therefore, HTSCs are extraordinarily sensitive to their microstructure demanding, at least, large grain and biaxially textured (epitaxial) films with clean grain boundaries, fully transparent for large values of electric current below T_c_ (Figure 1f). The HLH perovskites remain comparatively tolerant to microstructural requirements and occupy a position in between CMR and HTSC materials since they demand, due to an extraordinary large extinction coefficient, only 100–300 nm thin and uniform light absorbing layers with no pin holes to prevent shunting the circuit. This is demanded by the typical operational geometry of the new generation of solar cells with movement of negative and positive charge carriers across the sandwiched layers owing to the photovoltaic effect. The mean grain size of HTSC films would exceed millimeters (Figure 1f), the same typical parameter for CMR films falls into the submillimeter range (Pomerantseva et al., 2004). In contrast, HLH films with the best optoelectronic properties (Figure 1d) possess the grains of micrometer sizes and demand no perfect in-plain orientation although such films should contain no carrier traps at the boundaries (Zhang et al., 2016; Shlenskaya et al., 2018).

No real applications are known so far for single crystals of the noted perovskites although their effective grown techniques are developed successfully (Goodilin et al., 1997, 1998; Shiohara and Goodilin, 2000; Zhumekenov et al., 2018). Probably, some of them seem to be prospective for light-emitting devices, photodetectors and X-ray detectors (Wei and Huang, 2019; Murali et al., 2020). As for whiskers, they are not usually single crystalline, probably pseudomorphic (Petrov et al., 2017b), and are not yet involved in real applications.

HTSCs stand along among this group because of the second application domain related to large grain textured ceramics for industry like magnetic transport levitation, motors and generators (Tretyakov et al., 2004). In such ceramics, the same principle of biaxial texturing remains as in the case of HTSC thin films. Additionally, pinning centers of SC Abrikosov's vortexes are required for the ceramics however they are self-generated due to the developed processing schemes discussed below. At the same time, thin film solutions related to the so-called second generation of HTSCs are being developed to replace almost all high-current bulk materials.

Special requirements of achieving optimal morphologies lead to the wide elaboration of preparation techniques of these families of perovskite-related materials. In particular, thin film deposition and soft chemistry approaches are not unique and well-developed for all the discussing perovskite systems including, generally, different thermal, electron beam evaporation, laser ablation, CVD or MOCVD approaches, sol-gel and spin-coating techniques, aerosol spray pyrolysis, even graphoepitaxy; freeze drying is effective for preparation of some type of precursor particles (Tretyakov and Goodilin, 2000; Goodilin et al., 2002; Tretyakov et al., 2004; Gao et al., 2018; Snaith and Hacke, 2018; Nayak et al., 2019). Thin film deposition is quite complicated, but is already well-established, for the second generation of flexible tapes of HTSCs since this procedure includes unavoidably the metallic substrate texturing, coating with several buffer layers with precisely controlled microstructures followed by epitaxial growth of HTSC film, shunting and protective layers. The complexity of this procedure for finely tuned morphologies gave birth to special modifications of film deposition techniques like IBAD (ion beam assisted deposition) or ISD (inclined substrate deposition). Compared to that, HLH film deposition is much simpler and includes routine stages of deposition without controlling epitaxy relations. The specificity of those scale—up procedures for HLH is the ability to use nearly room temperatures and common solvents within the “wet” techniques like slot—die, blade-, spin-coating, ink–jet printing, screen printing etc (Li et al., 2018). Those methods have been surely tested for HTSC but they showed lower effectiveness compared to CVD or PVD because of much stricter requirement to the microstructure and higher phase conversion temperatures.

Solvent-deposition techniques of functional films seem to be oversimplified by common thinking however they are rather complicated by the phenomena of new phase formation, phase transformation, mass and heat transfer in terms of solvent evaporation, decomposition of intermediate hydrates and hydrolysis products (HTSC, CMR) or complex solvent adducts (HLH) resulting often in loosing morphological, chemical uniformity, shrinkage, crack formation etc (Petrov et al., 2017c; Shlenskaya et al., 2018). This all leads to the successful search for novel solvent-free, for example, melt-based, preparation approaches. Only two families of the perovskites utilize effectively melt preparation techniques—HTSCs and HLHs (Figure 1). In the first case, melt preparation is one of the basic and well-developed approaches (Table 1) while HLH phases have demonstrated such a potential only recently (Petrov et al., 2017a; Turkevych et al., 2019).

HTSCs undergo peritectic decomposition which can be exemplified for REEBa_2_Cu_3_O_7_ as “melting”: REEBa_2_Cu_3_O_7_ = REE_2_BaCuO_5_ + L + O_2_ where L–melt containing Cu(I) and Cu(2), REE_2_BaCuO_5_–a properitectic phase (Shiohara and Goodilin, 2000; Tretyakov and Goodilin, 2000). The reversal transformation depends on heat transfer, oxygen partial pressure and REE concentration in the melt. The latter is quite important because the melt is barium- and Cu(I)- rich and REE_2_BaCuO_5_ particles is the only source of REE. It actually gives no heterogeneous nucleation sites, as might be expected, it is proven that REE_2_BaCuO_5_, instead, provides a higher REE^3+^ content in the vicinity of the properitectic particles leading to homogeneous nucleation of REEBa_2_Cu_3_O_7_. Such a mechanism has direct consequences in terms of appearing several groups of melt processing (Table 1). All of these methods are already finally developed and therefore this system gives a full set of possible examples of effective melt preparation routes. In particular, most of the methods utilize, expectedly, melting and cooling regimes. Another group applies an isothermal controllable variation of pO_2_, as a volatile component, to crystallize the melt. Finally, some methods apply a spatial gradient of REE to provide the needed biaxial texture of HTSCs. The size distribution of the properitectic phase seems to be quite important since it determines the key morphological features of melt—processed large grain HTSC ceramics as well as the generation of effective pinning centers for type II superconductors. Therefore a special degree of freedom is used to vary this parameter closely connected with shifting the precursors toward more non-equilibrium states, in particular, by replacing the final product REEBa_2_Cu_3_O_7_ undergoing melting by imitators of the quenched peritectic melt, like REE_2_BaCuO_5_ and Cu(II) cuprate mixtures, or REE_2_O_3_ and Cu(I) cuprite mixtures, as observed upon decomposition of the REEBa_2_Cu_3_O_7_ phase at 1,300–1,400°C and quenching (Tretyakov et al., 2004). The best methods already known for HTSCs melt processing (Table 1) utilize both, the shift to non-equilibrium mixtures and the controllable change of pO_2_ (Tretyakov and Goodilin, 2000).

It is hard to expect that the HLH perovskites will undergo the same complex evolution of melt processing techniques since this family requires much simpler microstructures with no biaxial texturing, inclusions of secondary phases or large grains. At the same time, at least two analogous approaches are already suggested thus manifesting a start of development of HLH melt processing as a trend. Unfortunately, HLHs undergo irreversible decomposition with loosing highly volatile components like iodine and methylamine upon melting (Boyd et al., 2019). In this contest, traditional melt processing, even despite of comfortable melting points around 140–170°C is not applicable. At the same time, an excess of iodine or methylamine forms a self-flux - the room-temperature melts allowing crystallization of HLH perovskites from those liquids (Chen et al., 2017; Petrov et al., 2019). The recently developed and quite promising RP—MAGIC approach (Table 1) utilizes reactionary polyiodide melts (RPM) to convert thin layers of metallic lead to form a uniform film of light absorbing HLH: Pb + MAI_3_ = MAPbI_3_ (Turkevych et al., 2019). The driving force of this “chemical” crystallization process is that this is not an equilibrium system and has a huge difference in chemical potentials of components between the contacting phases. The chemical transformation of lead into a chemically compatible phase PbI_2_ results in its dissolution in RPM followed by crystallization of the HLH perovskite from supersaturated RPM since it is dictated by the driving force of the first stage of the lead interaction with RPM. Actually, this much resembles the most effective protocol of HTSC melt processing (OCMG, Tretyakov and Goodilin, 2000), starting from the REE_2_O_3_ phase incompatible with an admixture of Cu(I) barium cuprite which is transformed upon heating in liquid converting REE_2_O_3_ into the REE_2_BaCuO_5_ compatible phase followed by its dissolution in the cuprate melt under cooling to crystallize finally the REEBa_2_Cu_3_O_7_ HTSC (Tretyakov et al., 2004). Thus, the philosophy of preparation of new functional materials is common enough for different perovskites under the question with deviations naturally connected with chemical features predicted by the element's position in PTE.

## Conclusions and Perspectives

The families of promising perovskite materials discussed in this review have achieved quite different stages of implementation and practical applications. Despite the earliest discovery of CMR manganites, their applications are postponed for years because of their too narrow possible use in spintronics which is still not competitive with current trends in modern electronics. Expectations to use the CMR materials as wide-spread and fast magnetic sensors are moderate since SQUID devices outperform the manganites to a large extent. The remaining area of manganite applications is still connected with multiferroic systems and possible devices based on these multifunctional materials. HTSCs have achieved the heights of first industrial applications in superconducting electronics like SQUID and magnetic medical tomographs or industrial fault current limiters based on the second generation HTSC tapes, all after about 30 years beyond the HTSC discovery. There are a few already successful pilot projects of HTSC applications in transport systems like Maglev trains, propulsion ships and even small air craft jet systems. At the same time, a huge area of future applications of HTSCs is thinkable in megascience magnetic systems like synchrotrons, adroid colliders and thermonuclear plasma traps. However, the cost and operation stability issues still do not allow for the replacement of low temperature intermetallic superconductors. The frontier HLH solar cells and efficient light emitting devices, tandem power generating systems are the current challenges for science and engineering of hybrid perovskites with important remaining problems of stability and scaling up approaches. At the same time, their simpler architecture, chemistry and morphological requirements allow us to believe that these perovskites will come into commercial use much sooner compared to the other two families of perovskites, at least it is a prediction to optimistically believe.

## Author Contributions

EG prepared and wrote the manuscript draft. All authors added textual and reference information and approved the manuscript submission after mutual discussion.

## Conflict of Interest

The authors declare that the research was conducted in the absence of any commercial or financial relationships that could be construed as a potential conflict of interest.

## Footnotes

^1^Pearson, 1963; species with larger radii, corresponding smaller charge states and higher polarizability of chemical elements reduce the lattice energy of solid phases and their dependent “thermal stability.”
